# Draft Genome Sequence of *Amycolatopsis lurida* NRRL 2430, Producer of the Glycopeptide Family Antibiotic Ristocetin

**DOI:** 10.1128/genomeA.01050-14

**Published:** 2014-10-16

**Authors:** Min Jung Kwun, Hee-Jeon Hong

**Affiliations:** Department of Biochemistry, University of Cambridge, Cambridge, United Kingdom

## Abstract

We report here the first draft genome sequence for *Amycolatopsis lurida* NRRL 2430, the producer of the glycopeptide antibiotic ristocetin. The 9-Mbp genome is predicted to harbor 8,143 genes, including those belonging to the ristocetin biosynthesis cluster and 31 additional predicted secondary metabolite gene clusters.

## GENOME ANNOUNCEMENT

The glycopeptide family antibiotic ristocetin was originally used to treat Gram-positive pathogenic infections in humans, particularly staphylococcal infections. This use was soon discontinued, however, due to toxic side effects related to its ability to cause thrombocytopenia and platelet agglutination (1). Today, ristocetin is applied to the *in vitro* diagnosis of conditions, such as von Willebrand disease and Bernard-Soulier syndrome; blood from patients with the syndromes fail to exhibit a platelet agglutination response to ristocetin due to an absence of von Willebrand factor or its receptor (2). *Amycolatopsis lurida* is known to be the only producer of commercial ristocetin so far, but neither the whole-genome sequence information nor the sequence of its ristocetin synthetic gene cluster have been available. The sequence of the ristocetin biosynthesis gene cluster from *A. lurida* was recently published (GenBank accession no. KJ364518) (3).

The *A. lurida* NRRL 2430 genome was sequenced to 67-fold coverage using an Illumina MiSeq instrument, and it was assembled using the GS *de novo* Assembler (Newbler software version 2.9). A total of 1,993,479 reads were assembled into 101 contigs to produce a draft genome with an estimated size of 8,987,656 bp. The average contig size is 73,852 bp, and the largest single contig contains 424,186 bp. Annotation of the genome was performed using the NCBI Prokaryotic Genome Annotation Pipeline (4) with GeneMarkS+ (5). This annotation predicted 8,143 coding sequences (CDSs) with 109 pseudogenes, 54 tRNAs, two 5S rRNAs, one 23S rRNA, and an overall G+C content of 68.71%. The assembled draft genome sequence does not include any identifiable sequence for 16S rRNA, so we separately sequenced 16S rRNA in *A. lurida* NRRL 2430. Among several 16S rRNAs of different type strains of *A. lurida* available in the GenBank database, the 16S rRNA sequence from *A. lurida* NRRL 2430 perfectly matched one under the accession no. AJ577997 (6). According to the phylogenetic analysis of the 16S rRNA sequences, *A. lurida* NRRL 2430 is taxonomically most closely related to the strain *Amycolatopsis orientalis* HCCB10007, with 99% 16S rRNA identity. Analysis for secondary metabolite gene clusters using antiSMASH (7) identified 32 predicted clusters in the *A. lurida* NRRL 2430 genome, including the ristocetin synthetic gene cluster and clusters putatively responsible for producing terpenes, bacteriocins, type I, II, and III polyketide antibiotics, nonribosomal peptide synthetase (NRPS) compounds, ectoine, and several other unknown compounds

The genome information for *A. lurida* NRRL 2430 will help with understanding the mechanisms involved in the regulation of ristocetin biosynthesis, as well as assist the search for both new bioactive microbial metabolites and novel secondary metabolite-related enzyme activities.

### Nucleotide sequence accession numbers.

The draft genome sequence of *A. lurida* NRRL 2430 has been deposited in the DDBJ/EMBL/GenBank database under the accession no. JFBM00000000. The version described in this paper is the first version, JFBM01000000.
